# Prophylactic administration of miR-451 inhibitor decreases osteoarthritis severity in rats

**DOI:** 10.1038/s41598-022-20415-0

**Published:** 2022-09-27

**Authors:** Kayla M. Scott, D. Joshua Cohen, Dane W. Nielson, Gloria Kim, Lucas C. Olson, Michael J. McClure, Mark W. Grinstaff, Barbara D. Boyan, Zvi Schwartz

**Affiliations:** 1 College of Engineering, Virginia Commonwealth University, Richmond, VA USA; 2 Department of Biomedical Engineering and Chemistry, Boston University, Boston, MA USA; 3 Wallace H. Coulter Department of Biomedical Engineering, Georgia Institute of Technology, Atlanta, GA USA; 4 Department of Periodontics, University of Texas Health Science Center at San Antonio, San Antonio, TX USA; 5 Virginia Commonwealth University, 601 West Main Street (Suite 331), Richmond, VA 23284-3068 USA

**Keywords:** Osteoarthritis, miRNAs

## Abstract

Transfection of chondrocytes with microRNA-451(miR-451), present in growth zone cartilage of the growth plate, upregulates production of enzymes association with extracellular matrix degradation. miR-451 is also present in articular cartilage and exacerbates IL-1β effects in articular chondrocytes. Moreover, when osteoarthritis (OA) was induced in Sprague Dawley rats via bilateral anterior cruciate ligament transection (ACLT), miR-451 expression was increased in OA cartilage compared to control, suggesting its inhibition might be used to prevent or treat OA. To examine the prophylactic and therapeutic potential of inhibiting miR-451, we evaluated treatment with miR-451 power inhibitor (451-PI) at the onset of joint trauma and treatment after OA had developed. The prophylactic animal cohort received twice-weekly intra-articular injections of either 451-PI or a negative control (NC-PI) beginning on post-surgical day 3. OA was allowed to develop for 24 days in the therapeutic cohort before beginning injections. All rats were killed on day 45. Micro-CT, histomorphometrics, OARSI scoring, and muscle force testing were performed on samples. 451-PI mitigated OA progression compared to NC-PI limbs in the prophylactic cohort based on histomorphometric analysis and OARSI scoring, but no differences were detected by micro-CT. 451-PI treatment beginning 24 days post-surgery was not able to reduce OA severity. Prophylactic administration of 451-PI mitigates OA progression in a post-trauma ACLT rat model supporting its potential to prevent OA development following an ACLT injury clinically.

## Introduction

Osteoarthritis (OA) is a debilitating disease affecting over 300 million individuals worldwide characterized by cartilage degradation, subchondral bone remodeling, and joint pain^1^. Differing microRNA (miR) profiles have been found in diseased tissue compared to their healthy counterparts^2,3^, indicating microRNAs may be influencing disease development. miR-9, miR-27, miR-34a, miR-139, miR-140, and miR-146a are elevated in OA. Their expression is increased with interleukin-1 β (IL-1β) stimulation in vitro and they control cellular processes such as apoptosis and matrix metalloproteinase-13 (MMP-13) mediated degradation of articular cartilage extracellular matrix (ECM).

RNAseq analysis of rat costochondral cartilage growth plate chondrocyte cultures revealed miR profiles vary with developmental zone from which the cells were isolated^4,5^. miRs are packaged in matrix vesicles (MVs) produced by their parent cells and incorporated into their ECM^6^. miR-451a was specifically enriched^4,7^. When the growth plate chondrocytes were transfected with miR-451, production of enzymes associated with cartilage matrix degradation was increased^8^, suggesting it might also be involved in regulating the production and activity of these enzymes in articular cartilage.

Analysis of this microRNA using primary rat articular chondrocytes demonstrated that miR-451exacerbated IL-1β-induced increases in catabolic MMP-13 and inflammatory prostaglandin E2 (PGE2)^9^. Additionally, elevated levels of miR-451 expression were found in OA knees of Sprague Dawley rats 10 weeks following anterior cruciate ligament transection (ACLT) compared to sham knees^9^.

These data suggest that miR-451 may exacerbate OA progression. Conversely, inhibition of miR-451 might prevent or mitigate its severity. Therefore, the goal of this study was to assess the prophylactic and therapeutic potential of inhibiting miR-451 in an established rat ACLT model of OA by evaluating treatment at the onset of joint trauma and treatment after OA has developed. All animal studies were conducted in accordance with ARRIVE guidelines.

## Results

### 451-PI decreased evidence of OA when used prophylactically following ACLT

**Figure 1:**
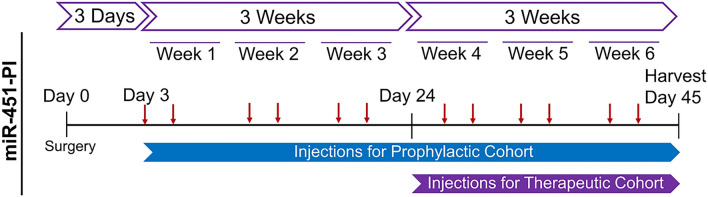
In-vivo experimental methods for miR-451-PI study. Eighteen Sprague Dawley rats underwent a bilateral ACLT surgery to induce OA. Two animals were used as controls (n = 4). The eighteen animals were split into two cohorts: 1) a prophylactic cohort that received intra-articular injections of miR-451-PI (n = 9) in left hind limbs or NC-PI (n = 9) in right hind limbs twice-weekly (red arrows) three days following surgery for a total duration of 6 weeks, and 2) a therapeutic cohort where we allowed OA to develop for 3 weeks before beginning the same injection regimen (n = 9 per treatment group, but one animal died resulting in an n = 8) for 3 weeks until animals were euthanized on day 42.

**Figure 2:**
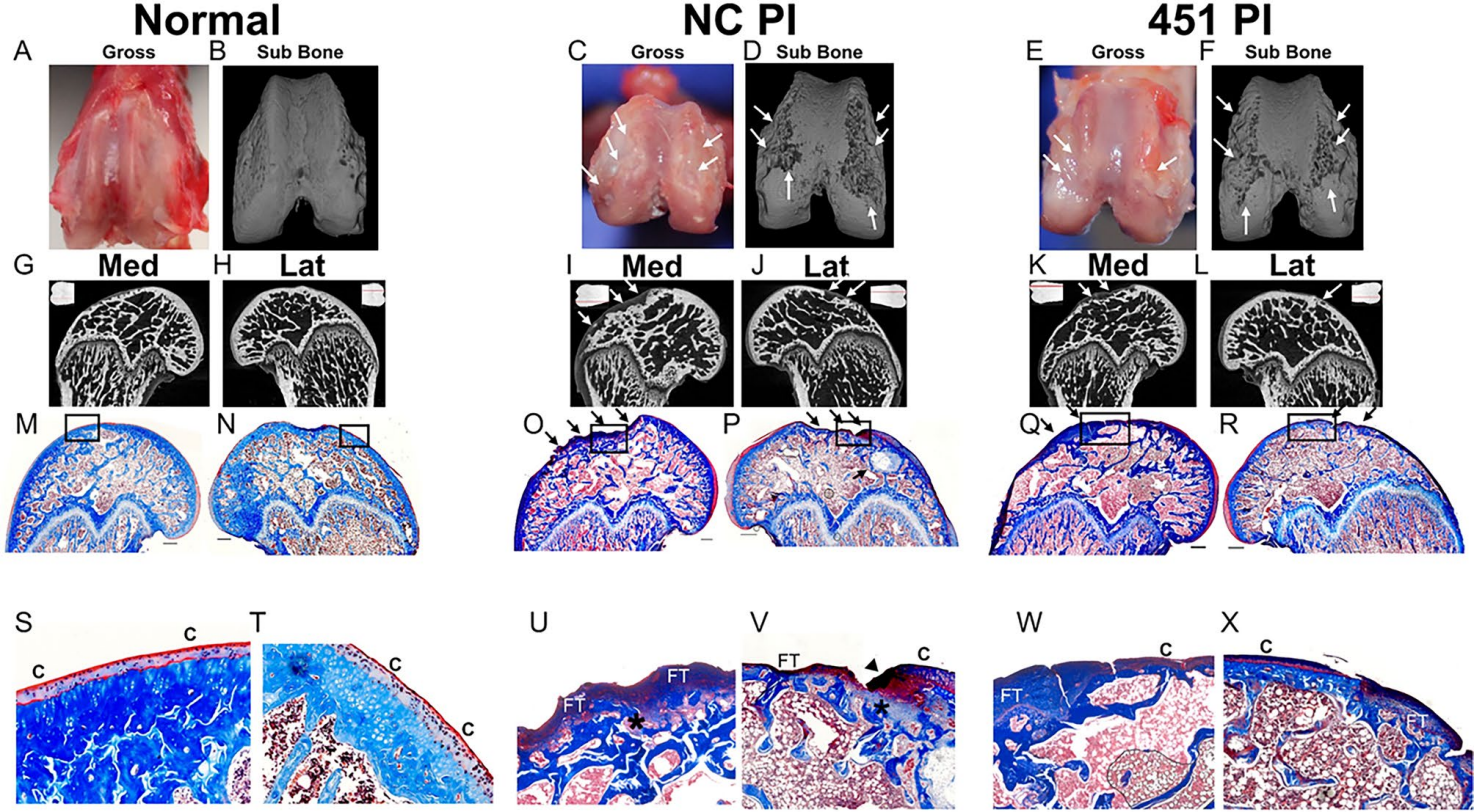
Gross images, micro-CT, and histology from the prophylactic cohort of the miR-451-PI study. Gross images (**A**,**C**,**E**), 3D micro-CT rendering (**B**,**D**,**F**), medial and lateral micro-CT images (**G**–**L**). A shadow projection of the femur is included in the top corner of each micro-CT image with a red line to indicate the location each micro-CT image is taken from within the joint (**G**–**L**). Masson’s trichrome histology (**M**–**R**) and the respective zoomed-in images as depicted by the black box (**S**–**X**) for the normal control, NC-PI, and 451-PI groups, respectively. Scale bar: 500 μm. Subchondral bone (sub bone); medial (med); lateral (lat); arrows, areas of cartilage erosion or subchondral bone remodeling; arrowheads, abnormal cartilage remodeling; C, cartilage; FT, fibrotic tissue; asterisk, microfracture.

Bilateral ACLTs were performed in 18 skeletally mature male Sprague–Dawley rats. Two rats were non-operated controls. For each study (prophylactic v. therapeutic), 9 rats were treated with miR-451 power inhibitor (451-PI) in their left leg and with a control molecule (NC-PI) in their right leg (Fig. 1). Gross images and 3D micro-CT reconstructions of femurs for control (Fig. 2A,B), NC-PI (Fig. 2C,D) and 451-PI legs (Fig. 2E,F) showed evidence of OA. Cartilage legions and subchondral bone erosion (white arrows) were present in NC-PI and 451-PI limbs as compared to control limbs. This was confirmed in micro-CTs of the medial and lateral femurs; normal aged controls did not have irregularities in the subchondral bone (Fig. 2G,H), whereas subchondral bone erosion and remodeling could be seen (white arrows) in the NC-PI injected knees (Fig. 2I,J), and 451-PI injected knees (Fig. 2K,L). Most of the damage to the ACLT joints was to the medial aspect, although some cartilage erosion was also evident on the lateral aspect. Masson’s trichrome stained medial and lateral femurs showed normal cartilage morphology in the controls (Fig. 2M,N), in contrast to the damaged articular cartilage (black arrows) in NC-PI (Fig. 2O,P), and 451-PI injected knees (Fig. 2Q,R).

At higher magnification, smooth articular cartilage surfaces (C) and healthy subchondral bone plates were seen in controls (Fig. 2S,T). Higher magnification of NC-PI (Fig. 2U,V) and 451-PI (Fig. 2W,X) limbs revealed abnormal cartilage remodeling (black arrowheads) and damage to the subchondral bone plate. There were microfractures and irregularities in the subchondral bone plate (asterisks), with more damage present in NC-PI limbs. Fibrotic tissue (FT) was present above the subchondral bone. This phenomenon has been reported when articular chondrocytes sustain damage, are unable to repair themselves, and de-differentiate into fibrotic chondrocytes^10^.

**Figure 3:**
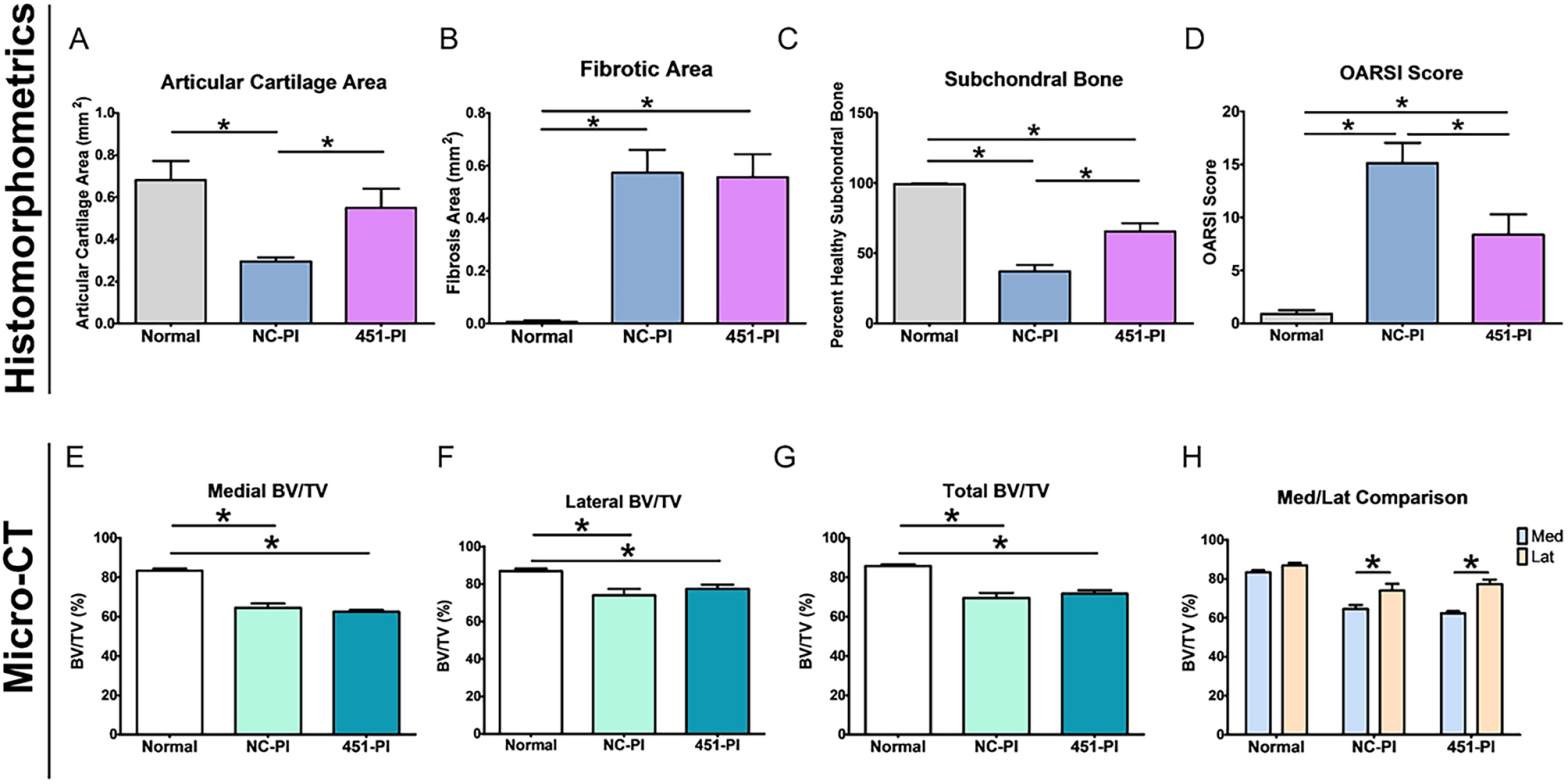
Prophylactic administration of 451-PI decreases OA severity. Histomorphometric analysis of articular cartilage area (**A**), fibrotic area (**B**), percent healthy subchondral bone (**C**), and OARSI scoring (**D**). Micro-CT bone volume/total volume (BV/TV) analysis of medial femurs (**E**), lateral femurs (**F**), total (medial + lateral, **G**), and the comparison of medial and lateral femurs (**H**). An asterisk indicates statistical significance using an alpha equal to 0.05.

Quantitative histomorphometry showed OA was present in both limbs that underwent bilateral ACLT surgery, regardless of treatment (Fig. 3A–C). Reduction in articular cartilage area was greatest in the NC-PI limbs (Fig. 3A). Fibrotic area in the ACLT legs was significantly increased compared to normal limbs with no difference between treatments (Fig. 3B). Subchondral bone quality was reduced in ACLT limbs, with effects on the NC-PI legs being greatest (Fig. 3C). Similarly, the OARSI scores in ACLT legs were significantly higher than in normal legs, and the OARSI score for NC-PI limbs was higher than for 451-PI limbs (Fig. 3D). Micro-CT analysis supported these findings. Medial BV/TV was reduced to a similar extent in ACLT limbs, with no differences evident due to treatment (Fig. 3E). This was also the case for lateral BV/TV (Fig. 3F) and for total BV/TV (Fig. 3G). Subchondral bone damage was greater in medial condyles in ACLT limbs (Fig. 3H), though both medial and lateral condyles were affected when compared to controls (Fig. 3E,F). Taken together, prophylactic treatment with 451-PI was able to reduce OA severity.

### 451-PI does not reverse OA progression

**Figure 4:**
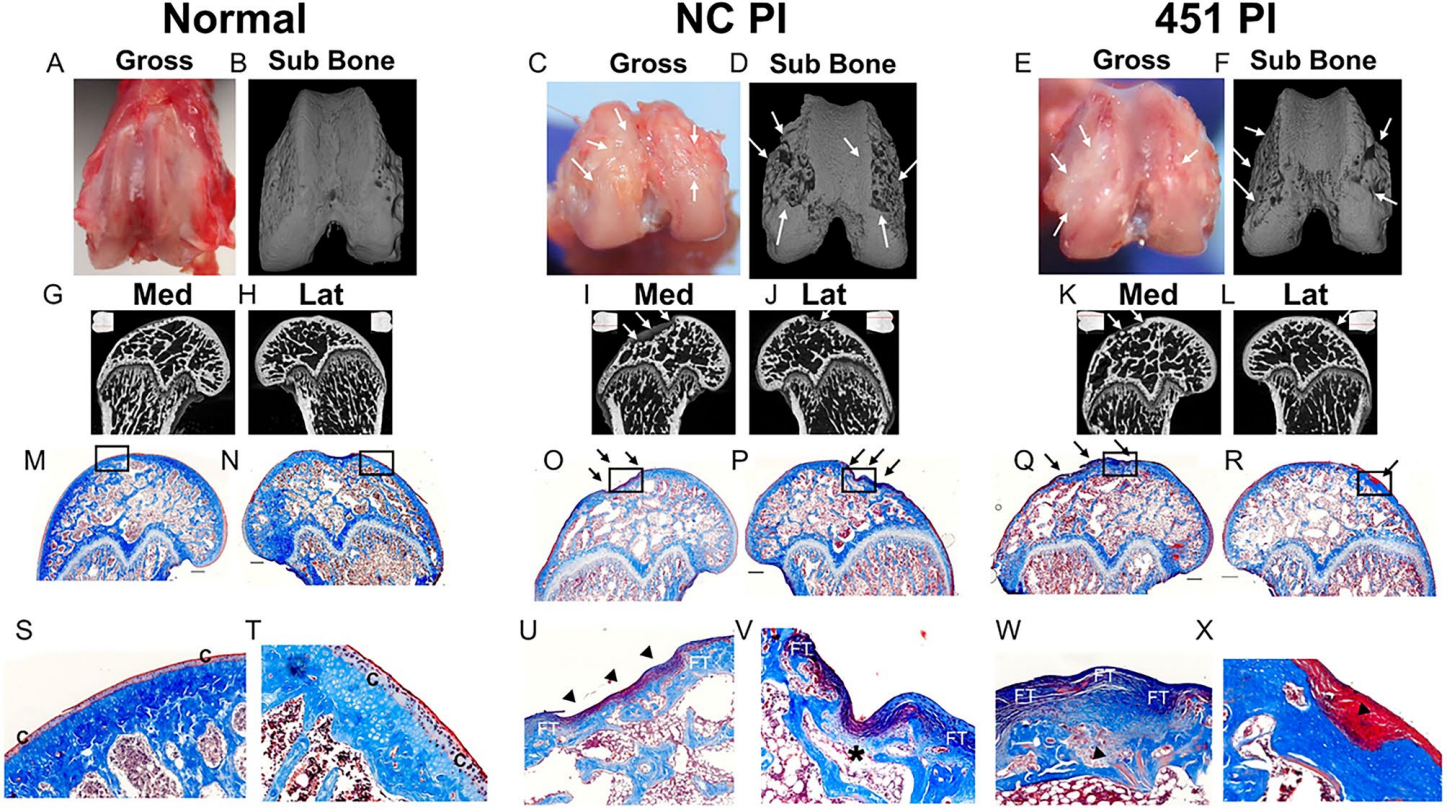
Gross images, micro-CT, and histology from the therapeutic cohort of the miR-451-PI study. Gross image (**A**,**C**,**E**), 3D micro-CT rendering (**B**,**D**,**F**), medial and lateral micro-CT images (**G**–**L**). A shadow projection of the femur is included in the top corner of each micro-CT image with a red line to indicate the location each micro-CT image is taken from within the joint (**G**–**L**). Masson’s trichrome histology (**M**–**R**) and the respective zoomed in images as depicted by the black box (**S**–**X**) for the normal control, NC-PI and 451-PI groups, respectively. Scale bar: 500 μm. Subchondral bone (sub bone); medial (med); lateral (lat); arrows, areas of cartilage erosion or subchondral bone remodeling; arrow heads, abnormal cartilage remodeling; C, cartilage; FT, fibrotic tissue; asterisk, microfracture.

When 451-PI injections were initiated on post-surgery day 24 and continued twice weekly for 3 weeks, there was no evidence that the damage caused by ACLT was reversed. Gross images and 3D micro-CT reconstructions of femurs of the control (Fig. 4A,B), NC-PI (Fig. 4C,D), and 451-PI (Fig. 4E,F) showed evidence of OA in ACLT limbs, demonstrated by cartilage legions and subchondral bone erosion (white arrows) in both NC-PI and 451-PI limbs. This finding was corroborated in micro-CTs of medial and lateral femurs. The controls had a healthy subchondral bone plate with no evidence of erosion (Fig. 4G,H). Conversely, the subchondral bone plate of NC-PI (Fig. 4I,J) and 451-PI (Fig. 4K,L) limbs showed evidence of erosion and remodeling (white arrows).

Masson’s trichrome stained medial and lateral femurs showed normal cartilage morphology in controls (Fig. 4M,N), while NC-PI (Fig. 4O,P) and 451-PI (Fig. 4Q,R) treated limbs exhibited cartilage damage and subchondral bone remodeling (black arrows). At higher magnification, smooth articular surface (C) and healthy subchondral bone plate was observed in controls (Fig. 4S,T). Higher magnification of NC-PI (Fig. 4U,V) and 451-PI (Fig. 4W,X) femurs revealed abnormal cartilage remodeling (black arrow heads), fibrotic tissue formation (FT), and microfractures (asterisks) in the subchondral bone. No obvious differences could be detected qualitatively in OA severity between NC-PI and 451-PI limbs.

**Figure 5:**
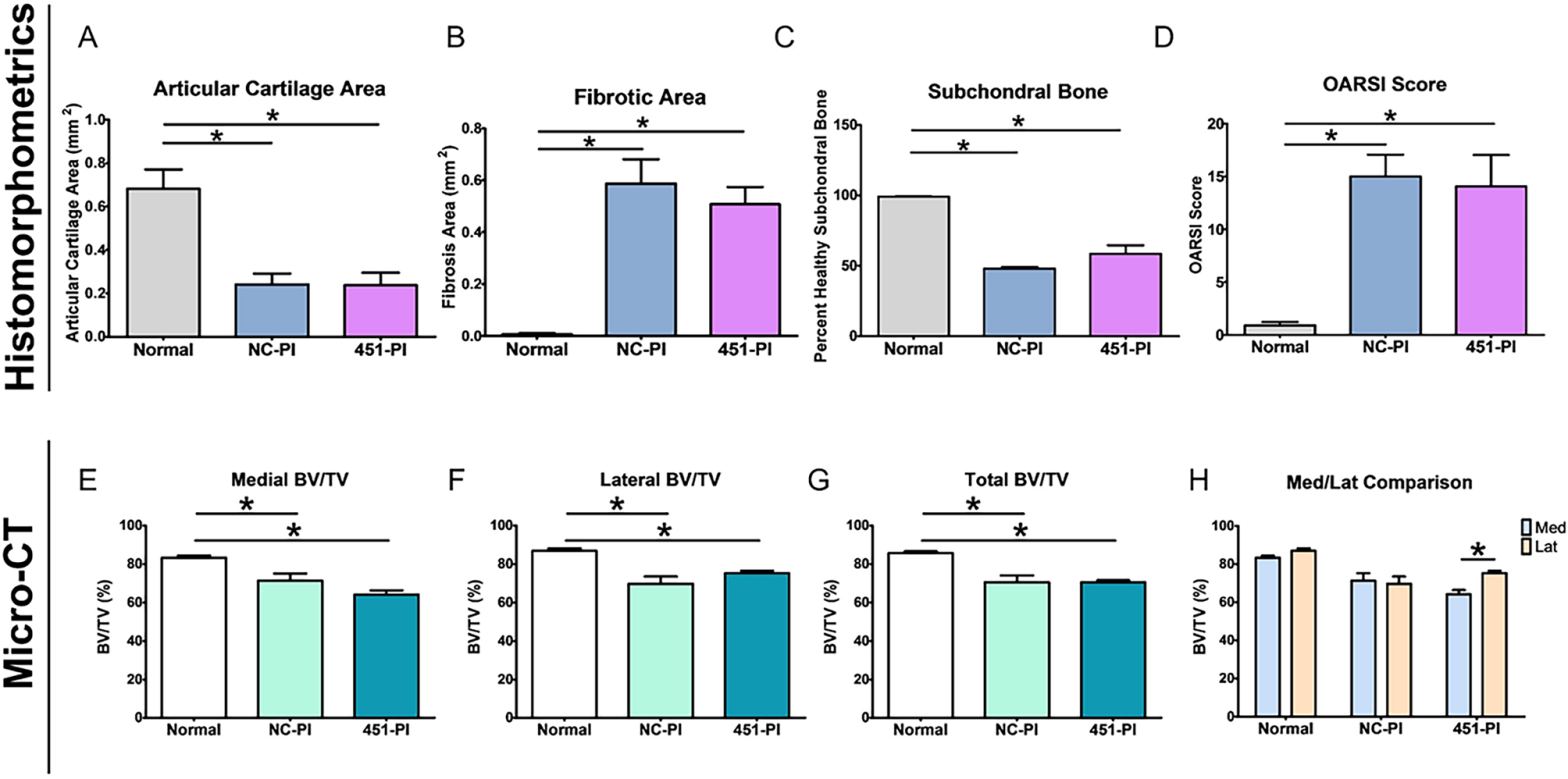
Therapeutic administration of 451-PI does not decrease OA severity. Histomorphometric analysis of articular cartilage area (**A**), fibrotic area (**B**), percent healthy subchondral bone (**C**), and OARSI scoring (**D**). Micro-CT bone volume/total volume (BV/TV) analysis of medial femurs (**E**), lateral femurs (**F**), total (medial + lateral, **G**), and the comparison of medial and lateral femurs (**H**). An asterisk indicates statistical significance using an alpha equal to 0.05.

Histomorphometry confirmed that OA was present following ACLT surgery, regardless of treatment. Both NC-PI and 451-PI treated limbs had decreased articular cartilage area (Fig. 5A), increased fibrotic area (Fig. 5B), decreased quality of subchondral bone (Fig. 5C), and increased OARSI scores (Fig. 5D) compared to controls. Histomorphometry indicated no differences between treatment limbs. Medial BV/TV was reduced to a similar extent in the ACLT limbs compared to controls (Fig. 5E). This was also the case for lateral BV/TV (Fig. 5F) and total BV/TV (Fig. 5G). Subchondral bone damage was greater in the medial condyle in the 451-PI treated limb, but not the NC-PI treated limb (Fig. 5H). OA did not affect force generation in the TA muscles as measured by maximum twitch and tetany in ACLT limbs compared to normal controls (Supplemental Fig. 4A–D). Taken together, therapeutic treatment with 451-PI did not reverse OA progression.

### Prophylactic treatment with 122-M does not mitigate OA progression

To validate our observations, in a second study we treated the rats with miR-122 mimic. We selected miR-122 for this analysis because it is also present in matrix vesicles produced by growth plate chondrocytes^4,8^ and is elevated in OA rat articular cartilage^9^, but it reduces the effects of IL-1 β on production of MMP-13 or PGE2 in rat articular chondrocytes^9^. When 122-M injections were given on post-surgery days 3, 12 and 21 (Supplemental Fig. 1), there was no apparent reduction in the severity of OA caused by ACLT. Gross images and 3D micro-CT reconstructions of femurs controls showed healthy cartilage and subchondral bone morphology (Supplemental Fig. 2A,B). In contrast, NC-PI (Supplemental Fig. 2C,D) and 122-M treated limbs (Supplemental Fig. 2E,F) exhibited cartilage legions and subchondral bone damage (white arrows). This was confirmed in micro-CTs of the medial and lateral femurs. No evidence of subchondral bone irregularities was seen in the normal aged controls (Supplemental Fig. 2G,H). However, ACLT limbs had evidence of subchondral bone remodeling and erosion (white arrows, Supplemental Fig. 2I,L).

Masson’s trichrome stained histology of the medial and lateral femurs showed a normal cartilage and subchondral bone morphology in controls limbs (Supplemental Fig. 2M,N), whereas there were abnormal cartilage remodeling (black arrow head), fibrotic tissue formation (FT) and microfractures in the subchondral bone plate (asterisk) in the NC-M (Supplemental Fig. 2O,P), and 122-M (Supplemental Fig. 2Q,R) treated limbs. At higher magnification, healthy cartilage (C) and normal subchondral bone plate were seen in the controls (Supplemental Fig. 2S,T). The higher magnification of NC-M (Supplemental Fig. 2U,V) and 122-M (Supplemental Fig. 2W,X) treated limbs displayed abnormal cartilage remodeling (black arrow head), fibrotic tissue formation (FT), and microfractures in the subchondral bone plate (asterisk). There was no discernable qualitative difference in OA severity between treatments.

Histomorphometry showed that OA was present in both limbs that underwent the bilateral ACLT surgery (Supplemental Fig. 3A–C). Both NC-M and 451-M treated limbs had decreased articular cartilage area (Supplemental Fig. 3A), increased fibrotic area (Supplemental Fig. 3B), decreased quality of subchondral bone (Supplemental Fig. 3C), and increased OARSI scores (Supplemental Fig. 3D) compared to controls. There were no differences between treatment limbs (Supplemental Fig. 3A–D). These data were corroborated by the micro-CT analysis. Medial BV/TV was reduced to a similar extent in the ACLT limbs compared to controls (Supplemental Fig. 3E). This was the case for lateral BV/TV (Supplemental Fig. 3F) and total BV/TV (Supplemental Fig. 3G). Subchondral bone damage was greater in the medial condyles in ACLT limbs (Supplemental Fig. 3H), though both medial and lateral condyles had decreased subchondral bone when compared to the control (Supplemental Fig. 3E,F). OA did not affect force generation in the TA muscles as measured by maximum twitch and tetany in ACLT limbs compared to normal controls (Supplemental Fig. 4E,F. Taken together, these data suggest that 122-M treatment was not able to mitigate OA disease progression when given with the current dosing regimen.

## Discussion

Increasing evidence indicates that microRNA play a role in disease pathology^11–17^, as well as osteoarthritis^18–20^. miR-140 attenuates early-stage OA progression by protecting against chondrocyte senescence^21^, while miR-204 and miR-211 ablation accelerates OA progression by increasing RUNX2 and osteogenic differentiation of mesenchymal progenitor cells^22^. Our research has shown that miR-451 is specifically increased in OA articular cartilage, although in vitro studies indicate that it does not induce apoptosis or compromise cell viability in chondrocytes^9,23^. However, in the presence of IL-1β, miR-451 exacerbates the production of catabolic MMP-13 and inflammatory PGE2^7^. This suggests that miR-451 cross-talks and synergistically stimulates downstream targets of the IL-1β signaling pathway that are only present after IL-1β stimulation, or in OA. Although the identities of these specific downstream targets were not clear using a TargetScan analysis^7^, the elevated presence of both IL-1β and miR-451 in OA make this a novel target for OA therapies^7^.

Our findings indicate that inhibiting miR-451 soon after injury and sustained over six weeks prevents OA progression in a severe bilateral ACLT model in rats. This observation was supported with histomorphometrics of the articular cartilage area, percent healthy subchondral bone and OARSI scoring. While early and sustained intra-articular injections using a specific miR-451 inhibitor was clearly beneficial, no differences were seen in fibrotic area covering the defect when compared to the contralateral limb treated with a control injection. This indicates that the target for miR-451 does not include pathways that regulate fibrosis.

Articular cartilage repair has been attempted in the last several decades using processes such as inhibiting or stimulating various cellular pathways^24–27^ and cell-based strategies using mesenchymal stem cells (MSCs) in cartilage lesions^28,29^. While improved outcomes have been demonstrated with these strategies, the most prominent issue is the development of fibrotic cartilage instead of hyaline cartilage^10^. Fibrocartilage has different mechanical properties and leads to detrimental outcomes in articular cartilage regeneration strategies^28^. In our study, inhibition of miR-451 did not significantly increase fibrocartilage generation above levels associated with OA.

Micro-CT analysis did not indicate that 451-PI reduced damage to the subchondral bone, but histomorphometric analysis showed that treatment with 451-PI was able to achieve subchondral bone closer to normal controls. Histomorphometry provided insight into the health and quality of the subchondral bone plate as determined by subchondral bone remodeling, micro-fractures, and osteophyte formation, whereas micro-CT indicated the total amount of bone present. Therefore, it is not surprising that there were differences in these metrics for the prophylactic cohort. The micro-CT analysis indicated a greater difference in subchondral bone plate in the medial femoral condyles compared to the lateral femoral condyles, as is supported by the literature for this model^30^.

Interestingly, miR-451 inhibition after OA onset (therapeutic cohort) did not appear to reduce OA severity. This may be due to the injection duration (3 total weeks of therapeutic injections as compared to 6 weeks), or to the fact that reversing OA after it has developed has never been achieved to-date. A study that evaluates a longer duration of 451-PI treatment after OA has developed may provide greater insight into miR-45-PIs therapeutic effects.

miR-122’s ability to prevent downstream effects of IL-1β stimulation in vitro made it an attractive option for treatment for OA^9^. miR-122 enhances sensitivity of hepatocellular carcinoma to the chemotherapy drug oxaliplatin by targeting WNT/β-catenin pathway^31^. An analysis of miR-122’s targets using the database TargetScan indicated that miR-122 may cross-talk with WNT/β-catenin signaling by targeting FOXO3 (Forkhead O3), FOXP2, and FOXK2^9^. The heavy involvement of WNT/β-catenin signaling in OA has been prevalent in the literature and studies have shown the activation of β-catenin signaling results in mouse chondrocytes undergoing phenotypical changes similar to that seen in OA^26^. Knockout of the extracellular WNT-signaling antagonist FrzB caused enhanced expression of MMPs and accumulation of β-catenin in chondrocytes stimulated with IL-1β^26^, which is supported by Frzb null mice having severe cartilage loss compared to wild type control mice in both post-traumatic and enzymatic models of OA^32^. Additionally, miR-122 may influence chondrocyte maturation and homeostasis markers such as SOX6 (sry-box transcription factor 6), IHH (Indian hedgehog), ADAM10 (ADAM metallopeptidase domain 10), and HIF3A (hypoxia-inducible factor 3-alpha)^9^. In our study, 122-mimic treatment did not mitigate the impact of ACLT, suggesting that its mode of action was not through a WNT/β -catenin pathway.

microRNAs are notoriously difficult to deliver in vivo. Over the last couple of decades, years of research have gone into therapeutic oligonucleotides, but only a handful have received market approval^33^. These work particularly well in vaccine development, as they need only present the oligonucleotide for a short period to initiate an immune response before they are destroyed. The most recent and well-known examples are the new immerging mRNA-bases vaccines for the ZVIKA, EBOLA, and SARS-CoV2 viruses. These lend value to the beneficial applications of oligonucleotide therapies, but highlight the current limitations with local administration, liver accumulation and rapid destruction^33^.

The introduction of chemical modifications such as locked nucleic acids (LNA) allowed better outcomes for microRNA inhibition delivery; however, the literature is still lacking widespread studies that deliver microRNAs mimics. Our 451-PI had specific proprietary chemical modifications that provided extra stability and allowed our inhibitor to enter the cell unaided through a process known as gymnosis. We used a different dosing regimen for the 122-M, and while there were modifications to the structure, they were not as robust as those to the inhibitor. Our results indicated that there was no difference in OA severity with 122-M treatment compared to NC. It is possible that 122-M was destroyed before it could elicit its effects, the dosing concentration was too low, or the dosing was too infrequent. This will need to be evaluated in future studies.

There were several limitations to our study. Different doses of both the 122 mimic and the 451 inhibitor weren’t examined to produce the optimal effect that may impact the therapeutic potential of the 451 inhibitor. The time of delivery needs to be further examined. We only examined immediately after injury, and four weeks post-injury when the OA was already established. Alternative delivery methods such as lipid nanoparticle should be examined to determine if there is increased efficacy. We did not add a fluorophore to our mimic to track location and persistence after administration. This would be a valuable option for future studies. Additionally, our injection design does not exclude the possibility of systemic effects from the treatment delivery vehicle, miR-122 mimic, or miR-451 inhibitor.

As part of this study, we characterized the surrounding muscle following ACL transection to determine if it impacted the development of OA. Muscle wasting due to decreased mobility and eventually compromised joint stability is hypothesized to impact OA progression^34^. Advanced hip and knee OA patients have lower limb muscle weakness and increased muscle atrophy^35–37^. There is a 10% reduction in the gastrocnemius area in OA rats following ACLT surgery^36^, which was similar to our findings, but no one has examined how the tibialis anterior muscle is affected in this model. We evaluated the twitch and tetany muscle force in the tibialis anterior in both studies and did not see any differences between any of the groups. Our results indicate that the tibialis anterior is not affected in contrast to the gastrocnemius, and its correlation to OA requires further examination^38–40^.

There are two critical periods when intervention may have the best outcomes for traumatic osteoarthritis. The first is immediately following injury to prevent the development of OA. The second is at the onset of clinical symptoms when cartilage damage has developed (active osteoarthritis). 80% of individuals who tear their ACL develop radiographic evidence of OA 5–15 years following injury^41^. Our results indicate that prophylactic administration of the miR-451 inhibitor may provide a clinical avenue for preventing OA following an ACL injury. However, our results indicate that the treatment of established osteoarthritis was not effective when 451-PI was administered after OA had developed.

## Materials and methods

### Rat anterior cruciate ligament transection (ACLT) model of OA

A total of thirty-one skeletally mature male Sprague–Dawley rats (Envigo, Indianapolis, IN), 9–10 weeks of age, weighing 250-300 g at study onset, were used for the two experiments. All animal procedures were performed according to the National Research Council’s Guide for the Care and Use of Laboratory Animals and approved by Virginia Commonwealth University’s Institutional Animal Care and Use Committee. In addition, all animal studies were conducted in accordance with ARRIVE guidelines. Animals were fed ad libitum. Based on a previous study^9^ using a 30% effect size, 20% variance, and a power of 80%, a two-tail analysis indicated an n = 8 per group was necessary to yield statistical significance.

### Treatment with 451-PI to prevent OA progression

The miR-451-PI study initially included twenty 9–10 week old male Sprague Dawley rats. Each treatment group had n = 8 plus 1 extra animal in case of morbidity or mortality. Nine rats were used to examine the prophylactic effect of 451-PI. Another nine animals were used to examine the therapeutic effect. Two animals were used as non-operated controls (n = 4 limbs). Rats were sedated 2–4% isoflurane anesthesia in 400 mL/min O2 to effect. Bilateral anterior cruciate ligament transections (ACLT, animals = 18) were performed by medially translocating the patellar tendon in order to visualize and transect the ACL to create a severe model of OA.

The rats were split into two cohorts, a prophylactic and a therapeutic cohort. In both cohorts, left hind legs received injections of rno-miR-451a (custom miRCURY LNA power inhibitor, 451-PI). Right hind legs received the negative control molecule (NC-PI). Both 451-PI and NC-PI were modified for in vivo use (PS, HPLC + Na + salt exchange) (Qiagen, Hilden, Germany). In order to evaluate the preventative potential of miR-451-inhibition, three days post-surgery, the prophylactic cohort (n = 9 rats, 9 legs/treatment group) received 50μL intra-articular injections of 100 nM of 451-PI and NC-PI twice a week, which was adapted from a previously published protocol^42^ (Fig. 1). Two animals (n = 4 limbs) were left untouched to serve as aged-matched controls and all four hind legs were used as controls (normal). The same controls were used for both cohorts in this study.

In order to evaluate the therapeutic potential of 451-PI, a therapeutic cohort was included that allowed OA to develop for 3 weeks before administering treatments at 3 weeks post-surgery for a total duration of 3 weeks. The therapeutic cohort was initially n = 9 rats (9 legs/treatment group). However, one animal died during surgery resulting in n = 8 legs/treatment group). All animals received the same dose and injection regimen as the prophylactic cohort, but treatment did not begin until day 24 rather than 3 days following surgery (Fig. 1).

Six weeks following the onset of treatment (post-surgery day 45), in vivo muscle force testing was conducted on the tibialis anterior (TA) while animals were sedated. Following muscle testing, animals were euthanized according to IACUC standards.

### Treatment with 122-M to prevent OA

Eleven 9–10 week old male Sprague Dawley rats were used to assess effectiveness of 122-mimic in preventing OA development. Eight animals underwent bilateral ACLT. Three days post-surgery, animals received 50μL intra articular injections of 2 mg/mL of either rno-miR-122 mimic 2.0 (HPLC purified, Life Technologies, Carlsbad, CA, 122-M), or rno-negative control #1. The injection leg (right vs left hind leg) was randomized for each animal (animals = 8, n = 8 legs/treatment group). Injection dose was modified from a previously published protocol^43^. Mimics were conjugated to the delivery vehicle invivofectamine 3.0 reagent (Thermo Fisher Scientific, Waltham, MA) according to the manufacture’s protocol. Injections were given on days 3, 12 and 21, for a total of three injections (Supplemental Fig. 1). A different injection technology was employed for this study; 122-M was delivered with invivofectamine whereas 451-PI was delivered naked using a modified structure that allowed entry into the cell via gymnosis. Based on the muscle physiology data generated in the first experiment, three animals were left untouched to serve as aged-matched controls and all six hind legs were used (normal). In vivo muscle force testing was conducted on the tibialis anterior (TA) while animals were sedated. Following muscle testing, animals were euthanized according to IACUC standards 30 days after surgery.

### MicroCT

Samples were fixed in 10% buffered formalin for 3 days. All scans were performed using a resolution of 2240 × 2240 (image pixel size of 7.91 μm), 45kVp, 177μA, 1300 ms exposure and rotation step size of 0.35 degrees^9^. Subchondral bone measurements were performed by drawing a region of interest (ROI) tightly around the subchondral bone plate. For areas where subchondral bone was remodeled and no longer present, the ROI was drawn as a projection of where the bone would be. The bone volume (BV) and total volume (TV) of the ROI was measured and a BV/TV percent was reported for the medial condyle (med BV/TV), lateral condyle (lat), and added together (total). MicroCT for the 451-PI study resulted in an n = 4 for controls, n = 9/treatment for the prophylactic cohort and an n = 7/treatment for the therapeutic cohort (one animal’s scan did not reconstruct properly due to corrupt files and one animal died during surgery). MicroCT for the 122-M study resulted in an n = 6 for the control and n = 8/treatment.

### Histology

Samples were decalcified in 40 mL 14% EDTA tetrasodium salt dehydrate (Millipore Sigma, Burlington, MA)^44^ containing 1.8% glacial acetic acid (Spectrum Chemical, Gardena, CA), pH 7.4–7.6, for 6–8 weeks. Solutions were changed twice weekly. Samples were rinsed in running deionized water for 15 min, dehydrated in a series of 70%, 95% and 100% ethanol and xylene washes, and embedded with Richard-Allan Scientific Histoplast Paraffin (Thermo Fisher Scientific). Sections (5 µm) were collected using a manual microtome (Shandon Finesse 325, Thermo Fisher Scientific) and stained using Masson’s trichrome. Samples were imaged using Zen 2012 Blue Edition software with an AxioCam MRc5 camera and Axio Observer Z.1 microscope (Carl Zeiss Microscopy, Oberkochen, Germany).

### Histomorphometrics

Articular cartilage and fibrotic area of medial femurs were measured using Zen 2012 Blue Edition software. The subchondral bone plate was measured and subdivided into healthy regions (normal cartilage above) and unhealthy regions (subchondral bone remodeling, osteophyte formation, and cartilage erosion/delamination). The percent healthy subchondral bone was reported as healthy subchondral bone divided by total subchondral bone *100. Histomorphometrics for the 451-PI prophylactic cohort study resulted in an n = 4 for controls, n = 9 for the NC-PI group and n = 8 for the 451-PI group due to histological processing errors. Histomorphometrics for the therapeutic cohort resulted in an n = 8/treatment. Histomorphometrics for the miR-122-M study resulted in an n = 5 for the control, n = 7 for the NC-M group and, n = 6 for the 122-M group due to histological processing errors.

### OARSI scoring

Two blinded reviewers scored histological samples and assigned a grade (0–6.5) to assess OA depth progression into the cartilage and a stage (0–4) to assess the horizontal extent of cartilage involvement along the articulating surface according to the OARSI scoring system guidelines^45^. Grade and stage were multiplied to obtain the OARSI score. The higher the score, the more severe OA present, with a max possible score of 26.

### Muscle force testing

Prior to euthanasia, in vivo muscle force testing was conducted on the tibialis anterior muscles using the 1300A Whole Animal System (Aurora Scientific, Canada). Animals were sedated (2–4% isoflurane/400 mL/min O2), and placed in a supine position on the platform. Each leg was immobilized at the knee using a screw-clamp and the paw was pressed firmly onto a pedal and secured with surgical tape. Two electrodes were inserted subcutaneously above the TA muscles to deliver electrical impulses for twitch and tetanic contractions. The foot flexes the pedal upon stimulation to produce a force–time curve. Twitch testing was performed 3 times using a 0.2 ms pulse width and the average of three test was reported. Tetany testing was performed 3 times at 140 Hz with a 500 m-sec stimulation duration until full muscle recruitment was reached followed by 120 s rest periods between stimulations to allow for muscle recovery. The average of three tests was reported. Muscle force testing was performed on all animals.

### Statistical analysis

Data are presented as mean ± standard error. The Grubbs’ test was used to determine statistical outliers using an alpha = 0.05. An asterisk indicates significant using a one-way analysis of variance followed by a two-tailed Tukey correction using an alpha = 0.05. All statistical analysis were performed using GraphPad Prism version 5.04 or JMP Pro 14.

## Supplementary Information

Supplementary Figure 1.

Supplementary Figure 2.

Supplementary Figure 3.

Supplementary Figure 4.

## Data Availability

The datasets generated during and/or analysed during the current study are available from the corresponding author on reasonable request.
