# Supplementary figures and images for: Prophylactic administration of miR-451 inhibitor decreases osteoarthritis severity in rats
Source: Sci Rep. 2022 Sep 27;12:16068. doi: 10.1038/s41598-022-20415-0 (PMC9513290; doi:10.1038/s41598-022-20415-0)

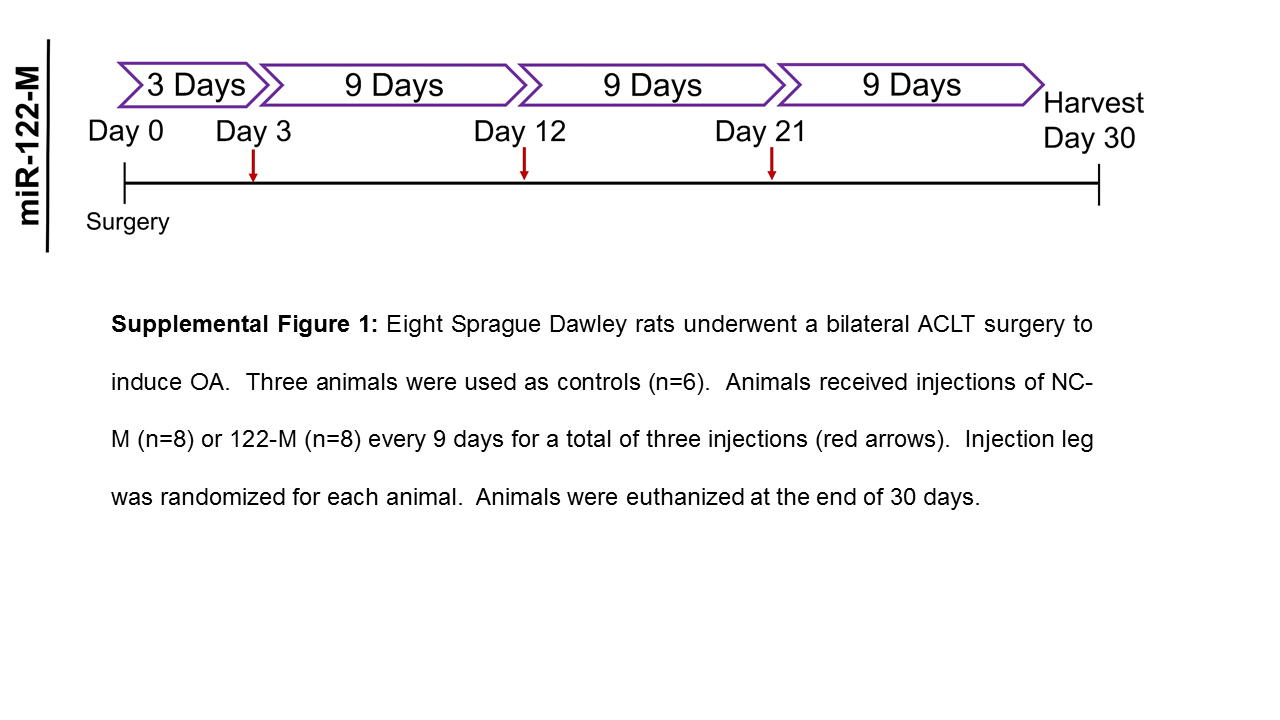

Supplement: Supplementary file 1 — Supplementary Figure 1. [file 41598_2022_20415_MOESM1_ESM.tif]

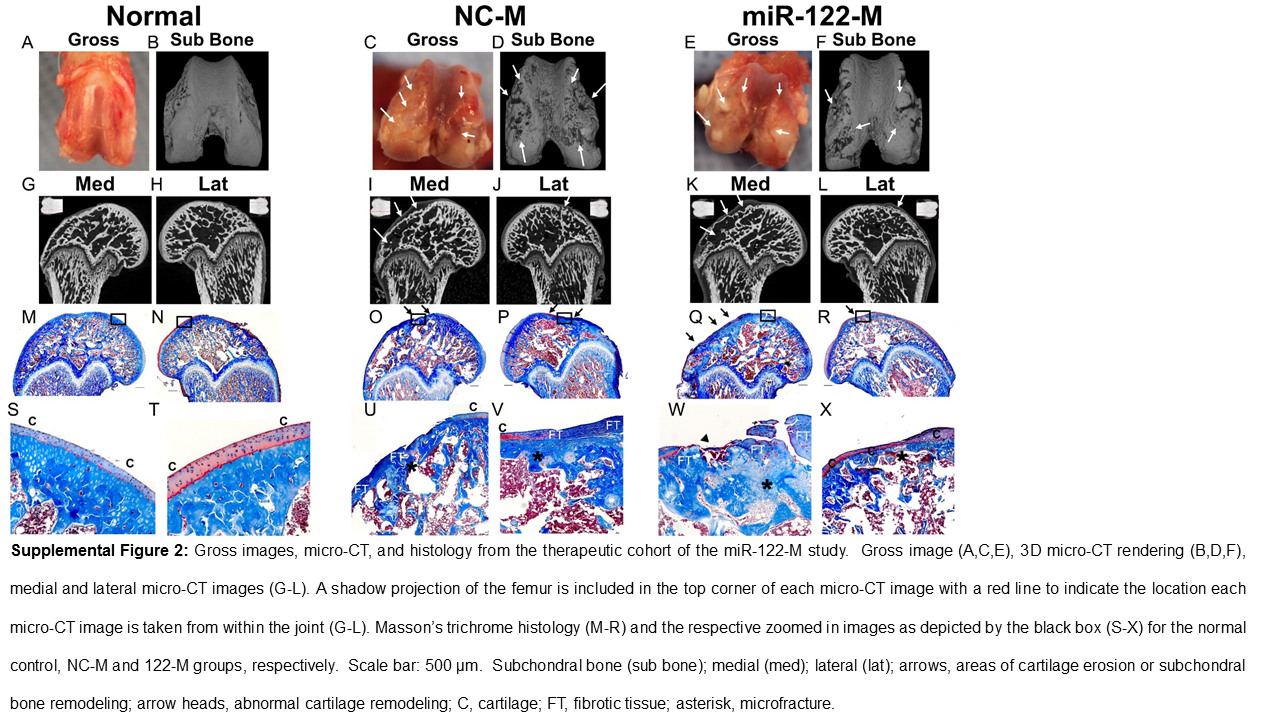

Supplement: Supplementary file 2 — Supplementary Figure 2. [file 41598_2022_20415_MOESM2_ESM.tif]

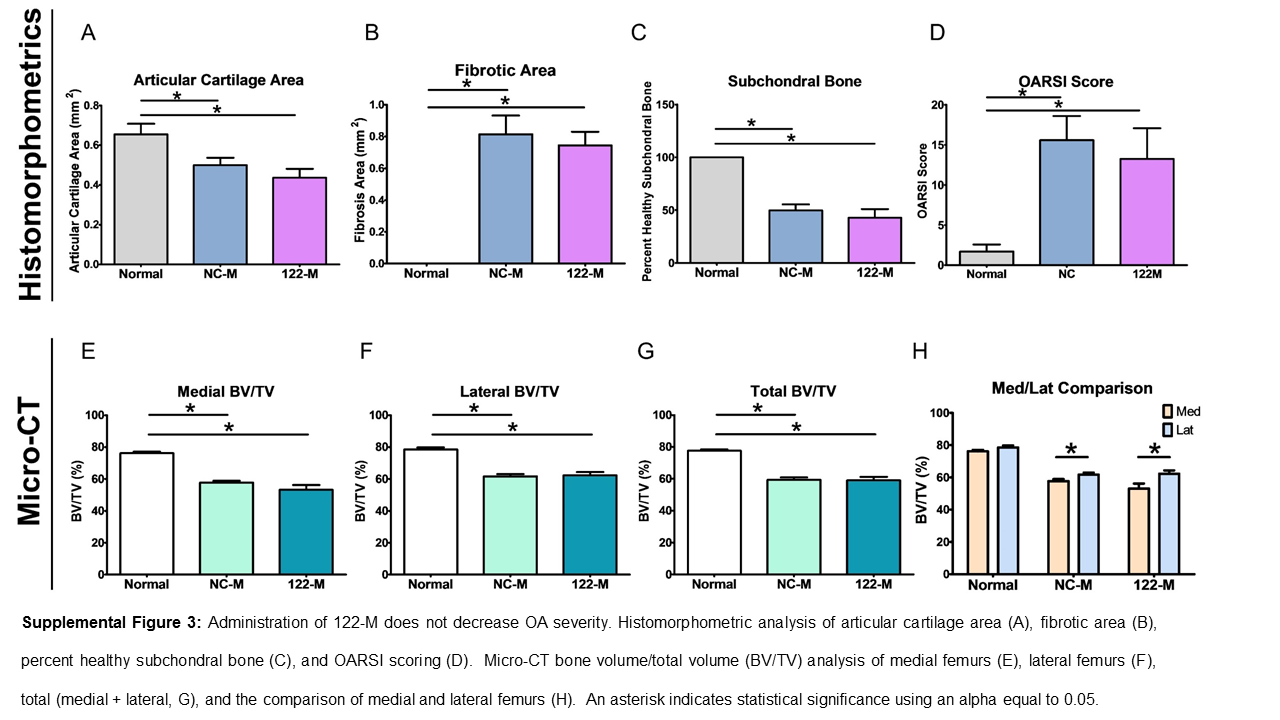

Supplement: Supplementary file 3 — Supplementary Figure 3. [file 41598_2022_20415_MOESM3_ESM.tif]

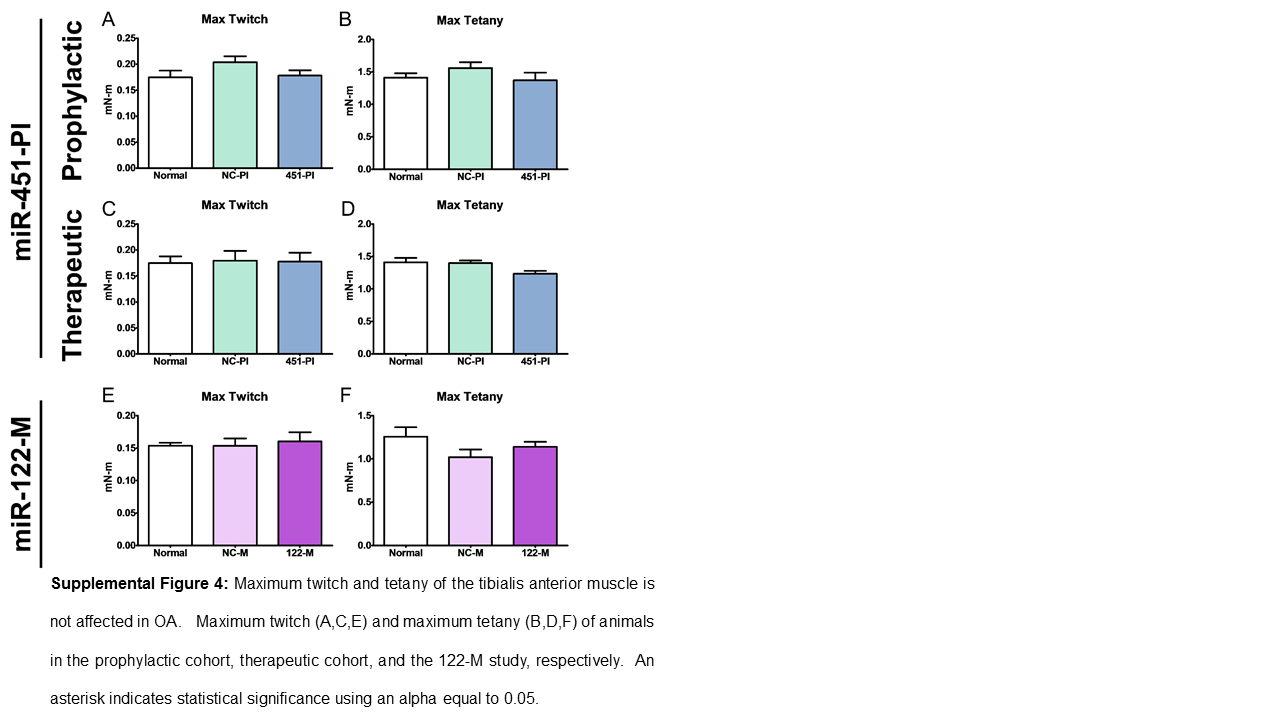

Supplement: Supplementary file 4 — Supplementary Figure 4. [file 41598_2022_20415_MOESM4_ESM.tif]
